# Subcutaneous pertuzumab–trastuzumab: Efficiency, safety, and the biosimilar horizon

**DOI:** 10.1016/j.breast.2025.104651

**Published:** 2025-11-19

**Authors:** Asim Armagan Aydin, Erkan Kayikcioglu

**Affiliations:** a Department of Clinical Oncology, University of Health Sciences, Antalya Education and Research Hospital, 07100, Antalya, Turkey; b Department of Medical Oncology, Istinye University School of Medicine, Bahcesehir Liv Hospital, 34517, Istanbul, Turkey

Dear Editor,

We read with great interest the article by Zietse and colleagues entitled *“Capacity and cost benefits of subcutaneous versus intravenous pertuzumab/trastuzumab: The EASE-SC study”* recently published in *The Breast* [1]. The authors provide a timely and comprehensive analysis of the operational and economic implications of switching from intravenous (IV) to subcutaneous (SC) fixed-dose pertuzumab/trastuzumab in patients with HER2-positive breast cancer. Their real-world micro-costing approach elegantly quantifies reductions in patient chair time, nursing workload, and non-drug expenditures offering valuable insights into how alternative administration routes can relieve oncology service bottlenecks amid global staffing shortages.

Yet, from a clinical and translational standpoint, several important aspects merit closer examination before these findings are generalized to practice or policy. First, while the study justifiably centers on cost and capacity metrics, it does not address the pharmacokinetic and immunogenic equivalence of SC versus IV regimens in real-world settings. Although phase III data (FeDeriCa trial) demonstrated non-inferior serum trough concentrations and comparable efficacy [2], post-approval evidence on injection-site reactions, systemic hypersensitivity, and local tolerability in routine practice remains sparse [3]. The rapid injection of two high-molecular-weight monoclonal antibodies within a single SC bolus may impose mechanical and immunologic stresses not captured in short-term observation windows. Prospective pharmacovigilance studies are warranted to ensure that efficiency gains do not inadvertently compromise safety or patient adherence, particularly when treatment is delivered outside tertiary centers.

Second, the cost-saving potential of SC administration must be viewed in the context of the evolving biosimilar landscape (*NCT05346224*) [4]. The impending market entry of IV pertuzumab biosimilars, projected within the next few years, will likely alter relative drug pricing and thereby reshape the economic calculus presented in the EASE-SC study [5]. As drug acquisition costs often dominate total expenditure, any differential advantage of the proprietary SC fixed-dose combination may diminish once biosimilar competition becomes widespread. Consequently, health-economic analyses should be dynamically updated to reflect these market shifts and avoid premature conclusions regarding long-term sustainability.

Third, the prospect of home-based or self-administered SC therapy, briefly alluded to by the authors, deserves cautious optimism. While decentralization of treatment could further enhance patient autonomy and reduce hospital burden, the oncologic context imposes distinct safety and compliance considerations compared with chronic immunologic diseases [6]. Regulatory guidance, education frameworks, and patient selection criteria will be critical prerequisites before such a paradigm can be safely implemented.

In summary, the EASE-SC study compellingly demonstrates the logistical and cost efficiencies achievable through SC HER2-targeted therapy. However, the clinical equivalence, pharmacovigilance oversight, and biosimilar-driven pricing dynamics remain key determinants of its real-world value. Integrating these dimensions into future analyses will provide a more holistic understanding of how SC biologic delivery can advance both efficiency and equity in modern breast cancer care.

## CRediT authorship contribution statement

**Asim Armagan Aydin:** Conceptualization, Investigation, Methodology, Supervision, Writing – original draft, Writing – review & editing. **Erkan Kayikcioglu:** Formal analysis, Methodology, Validation, Writing – review & editing.

## Funding statement

No specific grant from any funding agency in the public, commercial, or not-for-profit sectors was received for this work.

## Declaration of competing interest

The authors declare that they have no known competing financial interests or personal relationships that could have appeared to influence the work reported in this letter.
